# Paraformaldehyde Fixation May Lead to Misinterpretation of the Subcellular Localization of Plant High Mobility Group Box Proteins

**DOI:** 10.1371/journal.pone.0135033

**Published:** 2015-08-13

**Authors:** Man-Wah Li, Liang Zhou, Hon-Ming Lam

**Affiliations:** 1 Centre for Soybean Research of the Partner State Key Laboratory of Agrobiotechnology and School of Life Sciences, The Chinese University of Hong Kong, Shatin, Hong Kong; 2 Department of Radiation Medicine, School of Public Health and Tropic Medicine, Southern Medical University, Guangzhou, Guangdong, People’s Republic of China; German Cancer Research Center, GERMANY

## Abstract

Arabidopsis High Mobility Group Box (HMBG) proteins were previously found associated with the interphase chromatin but not the metaphase chromosome. However, these studies are usually based on immunolocalization analysis involving paraformaldehyde fixation. Paraformaldehyde fixation has been widely adapted to preserved cell morphology before immunofluorescence staining. On one hand, the processed cells are no longer living. On the other hand, the processing may lead to misinterpretation of localization. HMGBs from Arabidopsis were fused with enhanced green fluorescence protein (EGFP) and transformed into tobacco BY-2 cells. Basically, the localization of these HMGB proteins detected with EGFP fluorescence in interphase agreed with previous publications. Upon 4% paraformaldehyde fixation, AtHMGB1 was found associated with interphase but not the metaphase chromosomes as previously reported. However, when EGFP fluorescence signal was directly observed under confocal microscope without fixation, association of AtHMGB1 with metaphase chromosomes can be detected. Paraformaldehyde fixation led to dissociation of EGFP tagged AtHMBG1 protein from metaphase chromosomes. This kind of pre-processing of live specimen may lead to dissociation of protein-protein or protein-nucleic acid interaction. Therefore, using of EGFP fusion proteins in live specimen is a better way to determine the correct localization and interaction of proteins.

## Introduction

High mobility group (HMG)-box containing proteins are ubiquitous non-histone nuclear protein found in eukaryotes. The HMG-box proteins can be phylogenetically classified into four families including the chromosomal HMGB proteins, AT-rich interaction domain (ARID)-HMG proteins, 3xHMG-box proteins, and structure-specific recognition protein 1 (SSRP1). The chromosomal HMGB proteins can further be subdivided into three structurally distinct families including HMGA, HMGB, and HMGN [1]. The vertebrate HMGB proteins have two homologous DNA-binding motifs known as the HMG boxes (A and B) and an acidic C-terminal tail. They are able to recognize and bind preferentially to bent or distorted DNA structures such as four-way junctions (4H) [2], cisplatin-modified DNA [3], hemicatenated DNA loops [4], and supercoiled DNA [5]. The DNA binding capability of HMGB proteins also implies their involvement in DNA-dependent nuclear processes including transcription, DNA repairing and recombination.

A number of plant HMGB proteins have been cloned from Arabidopsis [6–8], rice [9], maize [10,11], and cotton [12]. These proteins were found involved in cell proliferation and differentiation [12], transcription [7,10], growth [13] and stress responses [12,13]. Unlike their vertebrate counterparts, these HMGB proteins contain only single HMG box.

Arabidopsis genome encodes 8 HMGB proteins. Some Arabidopsis HMGB proteins interact only with interphase chromatin but not the mitotic chromosomes [7,8,13]. It is also suggested that this characteristic can be a good indicator to distinguish HMGB proteins from the 3xHMG-box proteins which associates with condensed chromosome [1]. However, these studies commonly adopted indirect immunofluorescence method which involved fixation with paraformaldehyde [14,15]. Paraformaldehyde is widely used chemical fixative in immunohistochemistry and immunostaining because of its ability to preserve cell morphology by cross-link different biomolecules. Nevertheless, in an early study using animal cells, it has been suggested that paraformaldehyde fixation could affect the interaction between mammalian HMGB proteins and mitotic chromosome [16]. In this study, we examined whether the same artifact will occur in plant cells. Localization of AtHMGB1 protein in metaphase cells was reinvestigated using enhance green fluorescence protein (EGFP) tagged proteins with or without paraformaldehdye fixation.

## Materials and Methods

### Plasmid construction and tobacco cell transformation

Coding sequences of target genes were amplified from cDNA derived from *Arabidopsis thaliana* ecotype Columbia-0 seedlings with Platinum *Taq* DNA polymerase (10966–018, Invitrogen). The coding sequence of AtHistone H1.1 (AtH1.1; At1g06760), AtHMGB5 (At4g35570), AtHMGB12 (At5g23405), and AtHMGB14 (At2g34450) cloned into the *Xba*I site of a binary vector V7 [17] upstream of and in-frame with the CDS of EGFP. AtHMGB1 (At3g51880) was cloned into *Hin*dIII site of the same vector upstream of and in-frame with the CDS of EGFP. Wild type tobacco Bright Yellow (BY)-2 cells [18] was a gift from Prof. L. Jiang. Transformation and screening of the transgenic BY-2 cells were performed as previously described [19]. In brief, recombinant plasmids were transformed into *Agrobacterium tumefaciens* strain LBA4404. Four-day old wild type tobacco BY-2 cells were co-cultivated with the *A*. *tumefaciens* LBA4404 bearing the recombinant plasmids for 4 days in the present of 200 μM actosyringone at room temperature for 4 days. After washing 3 times with Murashige and Skoog medium in the present of 50 mg l^-1^ kanamycin and 250 mg l^-1^ cefotaxime, the cells were plated on Murashige and Skoog agar medium containing 50 mg l^-1^ kanamycin and 250 mg l^-1^ cefotaxime for selection.

### Confocal Microscopy

To analyze the effect of paraformaldehyde fixation, 4-day old transgenic BY-2 cells were treated with either phosphate-buffered saline (PBS) or 4% paraformaldehyde in PBS for 1 h with gentle shaking followed by two times PBS wash. For direct observation, cells were stained with 0.4 μg/ml Hoechst 34580 (H21486, Molecular Probes) in PBS for 15 min before confocal observation. Images were captured using Olympus FV1000 IX81-SIM Confocal Microscope with EGFP and 4',6-diamidino-2-phenylindole (DAPI) filter sets. After fixation, cells were digested with 0.1% cellulase (219466, Millipore) and 0.01% pectolyase Y23 (320951, MP Biomedicals) in PBS at room temperature for 15 min. After digestion, the cells were washed with PBS 3 times each for 5 min. Immunolabeling was carried out as previously described [7] with slight modifications. In brief, the cells were incubated in blocking solution (4% bovine serum albumin fraction V (BSA) and 0.1% Triton X-100 in PBS) for 1 h with gentle shaking. After washing twice with PBS, the cells were incubated overnight with 1:500 rabbit anti-GFP antibodies (A-11122, Thermo Fisher Scientific) in 1% BSA at 4°C. After washing twice with PBS, the cells were incubated with 1:500 goat anti-rabbit IgG (H+L) secondary antibodies, Rhodamine Red-X conjugated (R-6394, Thermo Fisher Scientific) in 3% BSA for 1 h at room temperature with gentle shaking. The cells were then washed with PBS twice, each for 15 min. Cells were mounted on slide with Vectashield mounting medium containing DAPI (H-1200, Vector Laboratories). Images were captured using Olympus FV1000 IX81-SIM Confocal Microscope with EGFP, DAPI and Rhodamine Red-X filter sets.

### Fluorescence recovery after photobleaching

Four-day old transgenic BY-2 cells were used for fluorescence recovery after photobleaching (FRAP) experiments with an Olympus FV1000 IX81-SIM Confocal Microscope. Photobleaching was performed using tornado mode with the 405 nm laser at 100% laser power for 0.6 s. EGFP fluorescence recovery was monitored with 488 nm laser using the free-run mode at ~3.9 s interval. Fluorescence of unbleached site in the same view was also monitored as the control. Signal was presented as the ratio relative to the fluorescence signal before photobleaching.

## Results and Discussion

### Establishment of transgenic tobacco cells for the study

**Fig 1:**
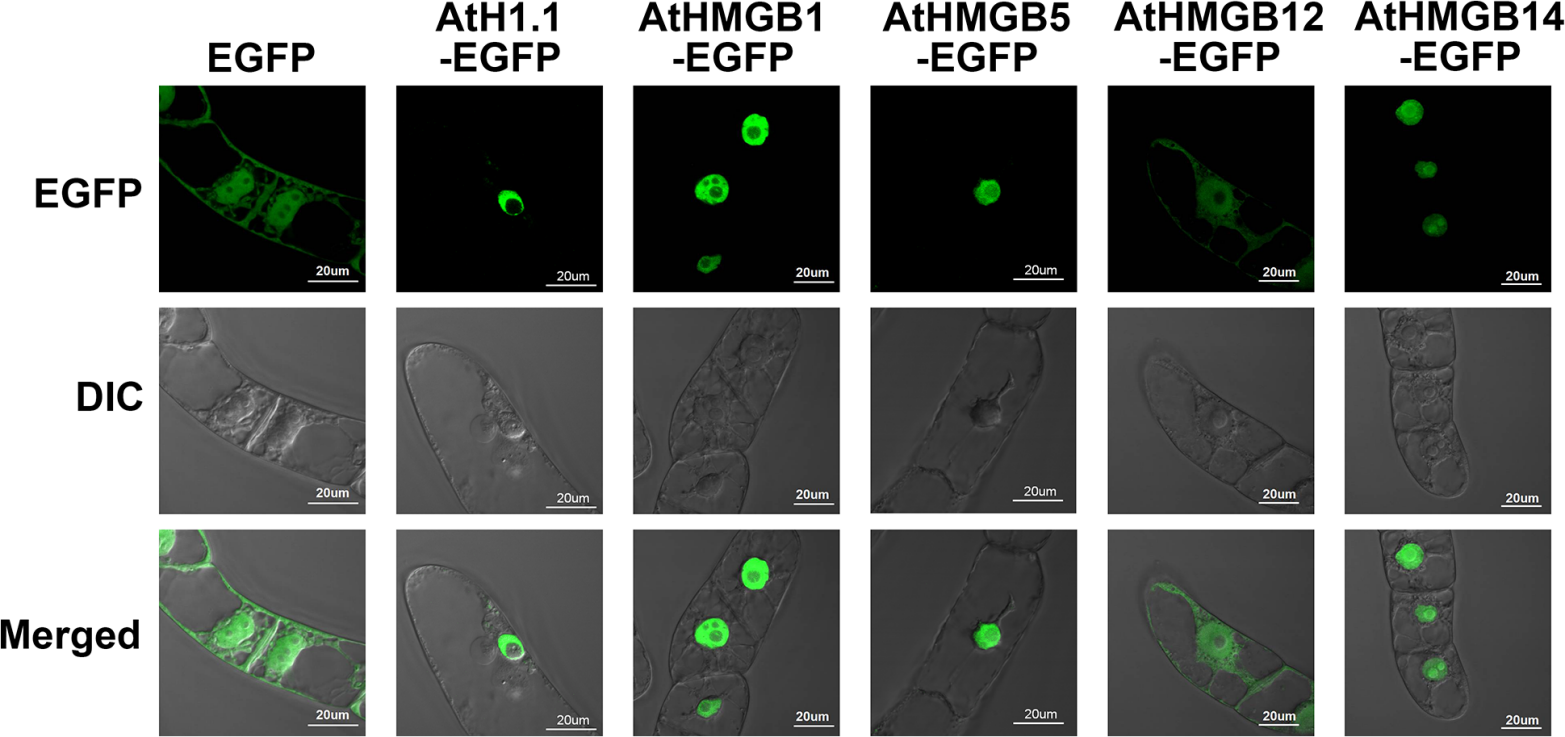
Interphase localization of EGFP tagged AtHMGB and AtH1.1 proteins. Live transgenic tobacco BY-2 cells expressing free EGFP, AtH1.1-EGFP, AtHMGB1-EGFP, AtHMGB5-EGFP, AtHMGB12-EGFP, and AtHMGB14-EGFP. Confocal microscope was used to study the localization of the EGFP tagged AtHMGB proteins at interphase without fixation. Top row: EGFP signal; middle row: Differential Interference Contrast (DIC); low row: merged image.

The cDNA clones of genes encoding AtHistone H1.1 (AtH1.1) and HMGBs were fused with the CDS of EGFP. The recombinant constructs were ectopically expressed in tobacco BY-2 cells for localization study. EGFP fluorescence of transgenic cells was observed directly under confocal microscope without fixation. During interphase, free EGFP localizes in both cytoplasm and nucleus (Fig 1). The histone protein AtH1.1 has strong binding with chromatin and the fluorescent signals of the fusion proteins was found only in nucleus but not the nucleolus (Fig 1). Fluorescence of EGFP fused to AtHMGB1, AtHMGB5 and AtHMGB14 were detected in nuclei and with diffused signal in the nucleolus (Fig 1). AtHMGB12 was detected in cytoplasm and nucleus resembling the free EGFP (Fig 1). Basically, the EGFP fused HMGB proteins imitated the localization as previously reported [6,7], suggested that the EGFP fusion does not lead to observable mis-localization.

### Paraformaldehyde fixation abolished the interaction between mitotic chromosome and AtHMGB1

**Fig 2:**
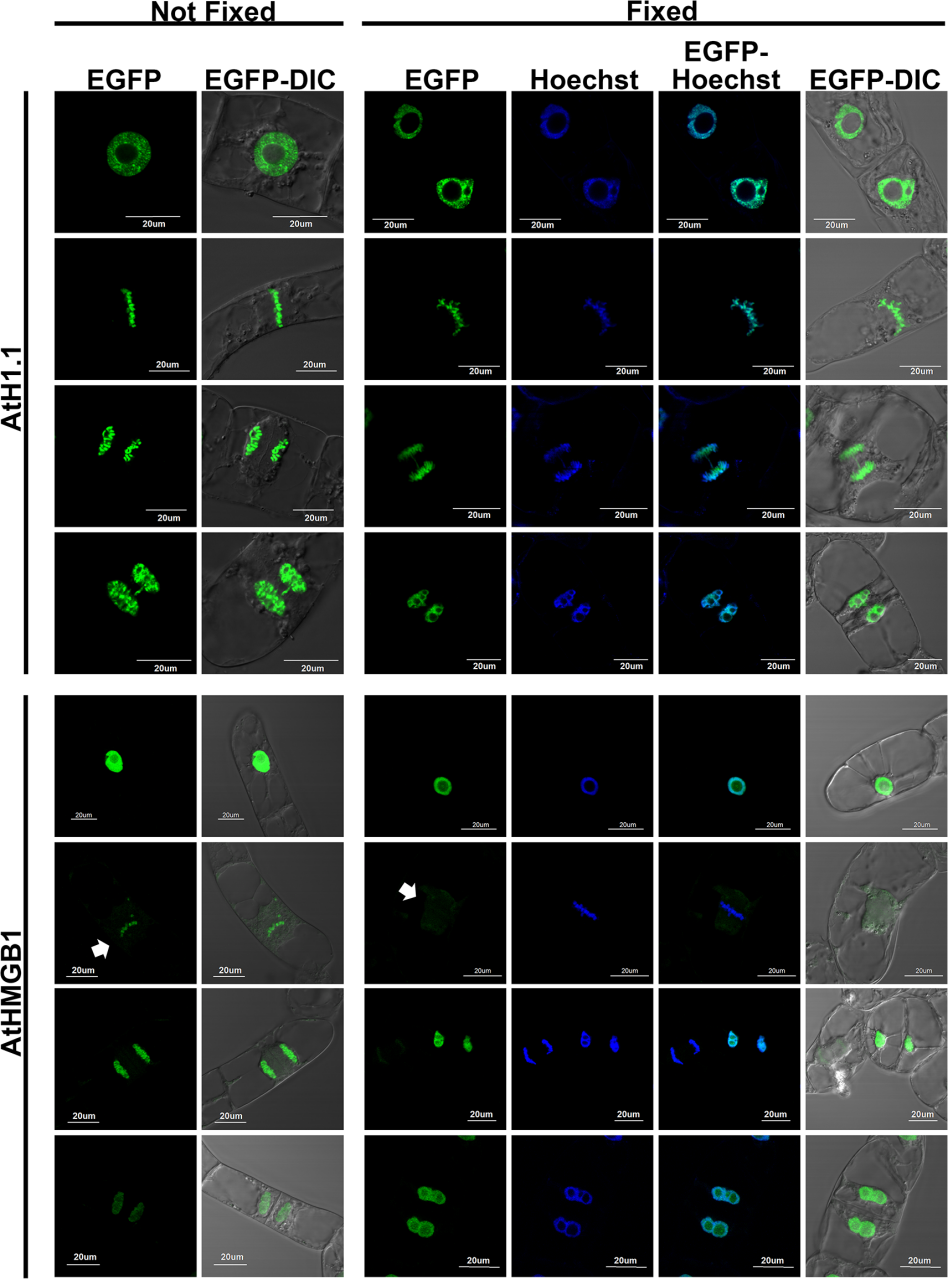
Localization of AtH1.1 and AtHMGB1 at different cell stages with or without paraformaldehyde fixation. EGFP signals of living unfixed (columns 1–2) and fixed (columns 3–6) transgenic tobacco BY-2 cells were detected using confocal microscopy. AtH1.1-EGFP interacted with chromosome at different cell stages with or without fixation (rows 1–4). AtHMGB1-EGFP in living cells interacted with chromosomes at different cell stages captured (rows 5–8). However the interaction between AtHMGB1-EGFP with mitotic chromosomes was abolished when the cells were fixed with paraformaldehyde. White arrow heads indicate the position of the metaphase chromosomes. Only one typical image of each cell stage was shown. Additional images can be found in S2–S5 Figs. A total of 10, 8, 9 and 6 metaphase chromosomes images of unfixed AtHMGB1, unfixed AtH1.1, fixed AtHMGB1 and fixed AtH1.1 were taken, respectively.

To further study the effect of paraformaldehyde fixation on the nuclear localization of AtHMGB proteins, we focus our study on the transgenic line expressing EGFP fusion of AtHMGB1. AtH1.1-EGFP was used as positive control to show that fixation does not lead to significant reduction of EGFP signal in nucleus (Fig 2, S1 and S2 Figs). Cells were fixed with 4% paraformaldehyde in PBS for 1 h without other additives. Another aliquot of cells were treated in PBS for the same period of time as the unfixed control. Hoechst 34580 was employed to stain the nuclear DNA of fixed cells. Hoechst 34580 could not penetrate the cell wall of live BY-2 cell and stained the cell wall heavily (S1 Fig).

With the Hoechst 34580 staining, it is observed that AtH1.1-EGFP signal is co-localized with the DNA staining in the fixed cells at all cell stages examined. The localization of AtH1.1 detected using EGFP fluorescence in fixed transgenic BY-2 cells resembled those of the unfixed cells (Fig 2). This suggested that EGFP fluorescence can be observed after paraformaldehyde fixation and the localization of AtH1.1 was not affected by fixation.

For the cells transformed with AtHMGB1-EGFP, there were discrepancies of AtHMGB1 localization between the unfixed and fixed cells. In interphase, there was no observable difference between the fixed and unfixed cells. In both cases, AtHMGB1 was detected to localize in nucleus and with diffused signals in nucleolus (Fig 2, S2 and S3 Figs). The compacted signals in the interphase nuclei co-localized with the Hoechst stain in fixed cells suggesting that AtHMGB1 interacts with the interphase chromosomes as previously reported [7]. However, the discrepancy occurred when comparing the fixed and unfixed metaphase cells. In the metaphase cells, although diffused signals could be detected in the cytoplasm, compacted EGFP signals were still detected along the metaphase plate in the unfixed AtHMGB1 cells (Fig 2).

**Fig 3:**
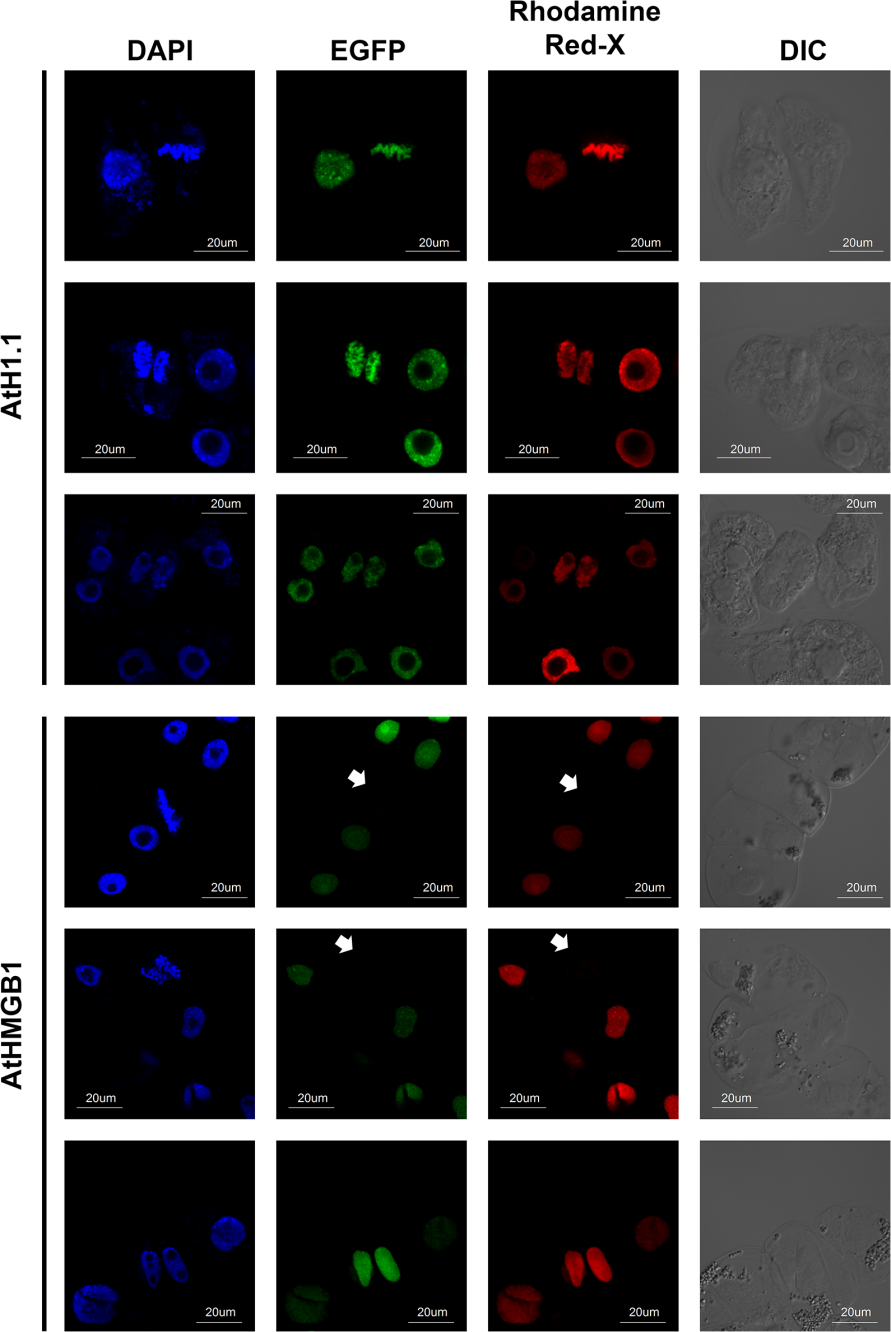
Localization of AtH1.1 and AtHMGB1 at different cell stages detected by immunolabeling. DAPI staining of chromosome (column 1), EGFP fluorescence signals (column 2), Rhodamine Red-X fluorescence signals (column 3) and DIC image (column 4) of transgenic tobacco BY-2 cells were detected using confocal microscopy. AtH1.1-EGFP interacted with chromosome at different cell stages with fixation (rows 1–3). The interaction between AtHMGB1-EGFP with mitotic chromosomes cannot be detected by both EGFP fluorescence and Rhodamine Red-X immunolabeled fluorescence upon fixation (rows 4–6). White arrow heads indicate the position of the metaphase chromosomes. Only one typical image of each cell stage was shown. Additional images can be found in S6 and S7 Figs. Eight images each of the metaphase chromosomes using AtHMGB1 or AtH1.1 antibodies were taken.

To demonstrate that the disappearance of AtHMGB1-EGFP signal on the metaphase chromosome was due to the change in binding of AtHMGB1 to metaphase chromosome but not due to the quenching or destruction of EGFP upon fixation, we performed further immunolabeling studies, using rabbit anti-GFP primary antibodies and Rhodamine Red-X conjugated goat anti-rabbit IgG secondary antibodies. Consistent to the results of the above confocal microscopic studies, Rhodamine Red-X fluorescence can be detected in interphase nuclei and metaphase chromosomes of transgenic BY-2 cells expressing AtH1.1-EGFP (Fig 3, S7 Fig). On the contrary, Rhodamine Red-X fluorescence can be detected in interphase nuclei but not the metaphase chromosomes of transgenic BY-2 cells expressing AtHMGB1-EGFP (Fig 3, S6 Fig). This result supports our notion that paraformaldehyde fixation could lead to a misinterpretation of AtHMGB1 localization.

**Fig 4:**
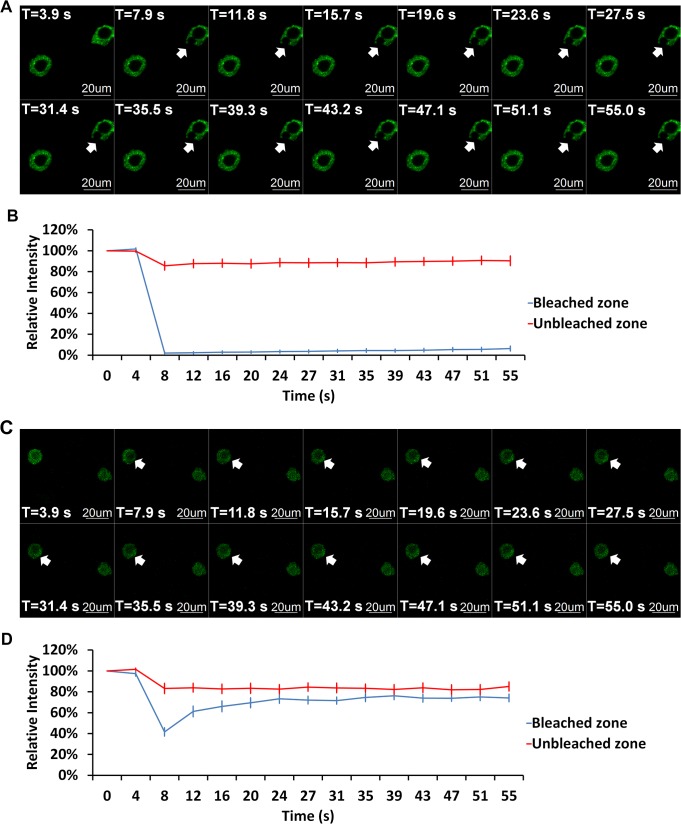
FRAP analysis of AtH1.1 and AtHMGB1 in prophase nuclei. (A) FRAP images of AtH1.1-EGFP transgenic BY-2 cells. (B) FRAP recovery curves of AtH1.1-EGFP. (C) FRAP images of AtHMGB1-EGFP transgenic BY-2 cells. (D) FRAP recovery curves of AtHMGB1-EGFP. For (B) and (D), blue lines showed the relative signal of the bleached zones. Bleach started at the 3.39^th^ second and ended at 3.99^th^ second. Red lines showed the relative signal of the unbleached zones. Bleaching sites were marked with white arrow heads. Each curve represented the average of 9 and 10 separate FRAPs analysis of AtH1.1 and AtHMGB1, respectively. Error bar: standard error.

It is suggested that AtHMGB1 does interact with metaphase chromosomes which align along the metaphase plate in living cells. The diffused signal was probably due to the highly dynamic interaction between AtHMGB1 and chromosomal DNA [7]. Fluorescence recovery after photobleaching (FRAP) analysis can be used to study interaction in living cells [18]. FRAP recoveries require dynamic diffusion of fluorescent molecules. FRAP study of EGFP tagged AtHMGB1 and AtH1.1 in transgenic BY-2 cells at interphase suggested that AtHMGB1 dynamically replenished the fluorescence upon photobleaching while AtH1.1 did not (Fig 4). Without the confinement of nuclear membrane during the metaphase, the highly mobile AtHMGB1 was free to diffuse into the cytoplasm and hence diluted the local concentration of AtHMGB1 proteins in the “nucleus” and led to reduction of signals there. However, this observable interaction between AtHMGB1 and DNA was abolished upon fixation. Only diffused EGFP signal was detected in fixed AtHMGB1 metaphase cells, but not along the metaphase plate as indicated by the Hoechst 34580 or DAPI signals.

Chemical fixations have long been found to be problematic for microscopic studies [20]. They may create artifacts due to the disruption of cellular structures or lowering of protein antigenicity [20]. Fixation through high pressure freezing, freeze substitution and resin embedding [20,21] have been suggested to be a better way to preserved the cellular structure during electron microscopic studies. Confocal microscopy can also be done using samples embedded in resin though reactivation of fluorophore may be needed [22,23].

## Conclusions

EGFP fusion of HMGB proteins from Arabidopsis can imitate the localization of these proteins at interphase detected by immunolocalization study. However, paraformaldehyde fixation led to dissociation of AtHMGB1-EGFP fusion protein from metaphase chromosomes suggesting that pre-processing of live specimen with paraformaldehyde may lead to dissociation of protein-protein or protein-nucleic acid interactions and lead to misinterpretation of the actual localization. Our observation suggested that detection of EGFP fusion proteins using confocal microscope would be a better way to determine the localization of HMGB protein in mitotic cells.

## Supporting Information

S1 Fig Hoechst 35480 stain cannot stain the chromosomes of live tobacco BY-2 cells. A typical photo showing that Hoechst 35480 stain cannot stain the chromosomes of live tobacco BY-2 cells. While the cell wall was heavily stained by Hoechst 35480, this dye could not penetrate into the cell to stain the chromosomes.
(TIF)

S2 Fig Some typical confocal images of unfixed AtHMGB1 transgenic BY-2 cells. Odd columns showed the EGFP images and the even columns showed the DIC images.
(TIF)

S3 Fig Some typical confocal images of paraformaldehyde fixed AtHMGB1 transgenic BY-2 cells. Columns 1 and 4 showed the Hoechst staining images, columns 2 and 5 showed the EGFP images, and columns 3 and 6 showed the DIC images.
(TIF)

S4 Fig Some typical confocal images of unfixed AtH1.1 transgenic BY-2 cells. Odd columns showed the EGFP images and the even columns showed the DIC images.
(TIF)

S5 Fig Some typical confocal images of paraformaldehyde fixed AtH1.1 transgenic BY-2 cells. Columns 1 and 4 showed the Hoechst staining images, columns 2 and 5 showed the EGFP images, and columns 3 and 6 showed the DIC images.
(TIF)

S6 Fig Some typical confocal images of paraformaldehyde fixed AtHMGB1 transgenic BY-2 cells. Columns 1 and 5 showed the DAPI staining images, columns 2 and 6 showed the EGFP images, columns 3 and 7 showed the Rhodomine Red-X images, and columns 4 and 8 showed the DIC images.
(TIF)

S7 Fig Some typical confocal images of paraformaldehyde fixed AtH1.1 transgenic BY-2 cells. Columns 1 and 5 showed the DAPI staining images, columns 2 and 6 showed the EGFP images, columns 3 and 7 showed the Rhodomine Red-X images, and columns 4 and 8 showed the DIC images.
(TIF)
