# Supplementary figures and images for: Paraformaldehyde Fixation May Lead to Misinterpretation of the Subcellular Localization of Plant High Mobility Group Box Proteins
Source: PLoS One. 2015 Aug 13;10(8):e0135033. doi: 10.1371/journal.pone.0135033 (PMC4535772; doi:10.1371/journal.pone.0135033)

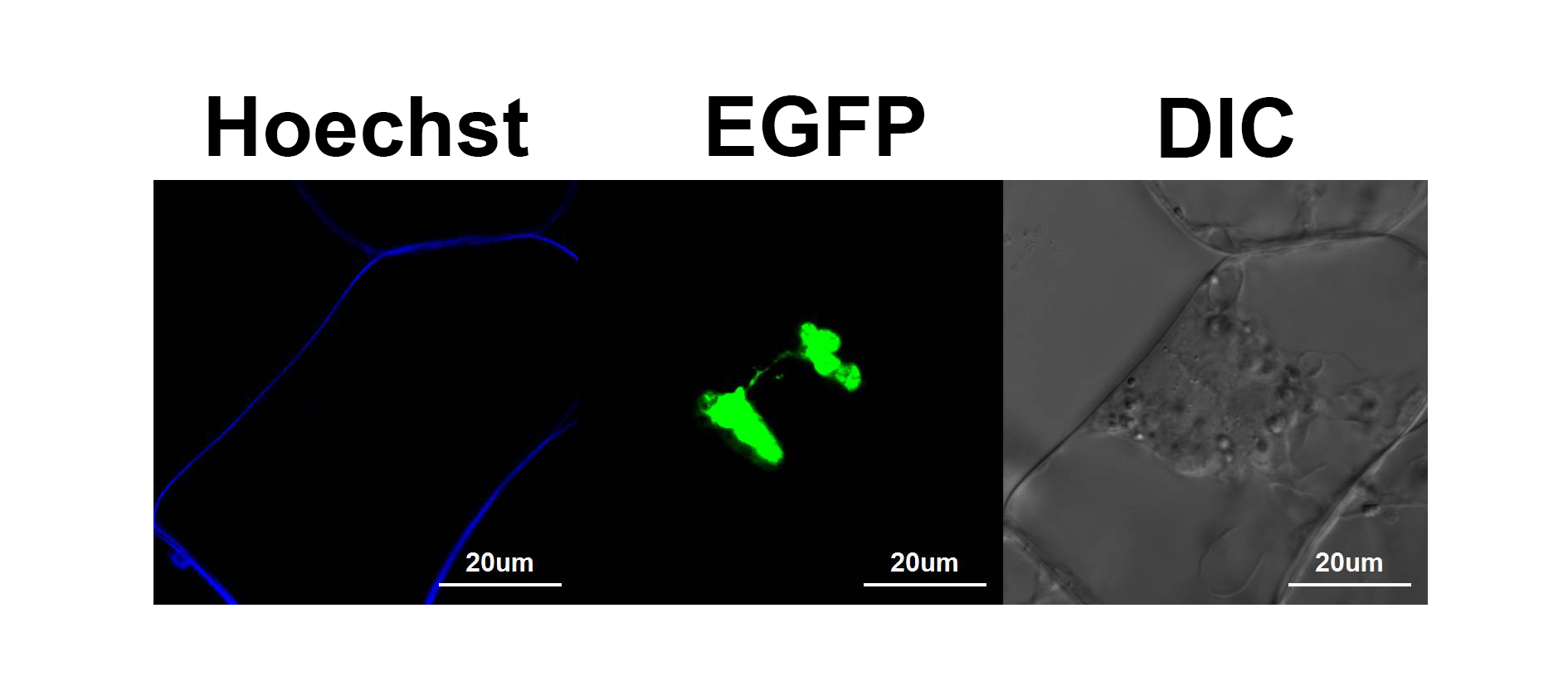

Supplement: S1 Fig — A typical photo showing that Hoechst 35480 stain cannot stain the chromosomes of live tobacco BY-2 cells. While the cell wall was heavily stained by Hoechst 35480, this dye could not penetrate into the cell to stain the chromosomes. (TIF) [file pone.0135033.s001.tif]

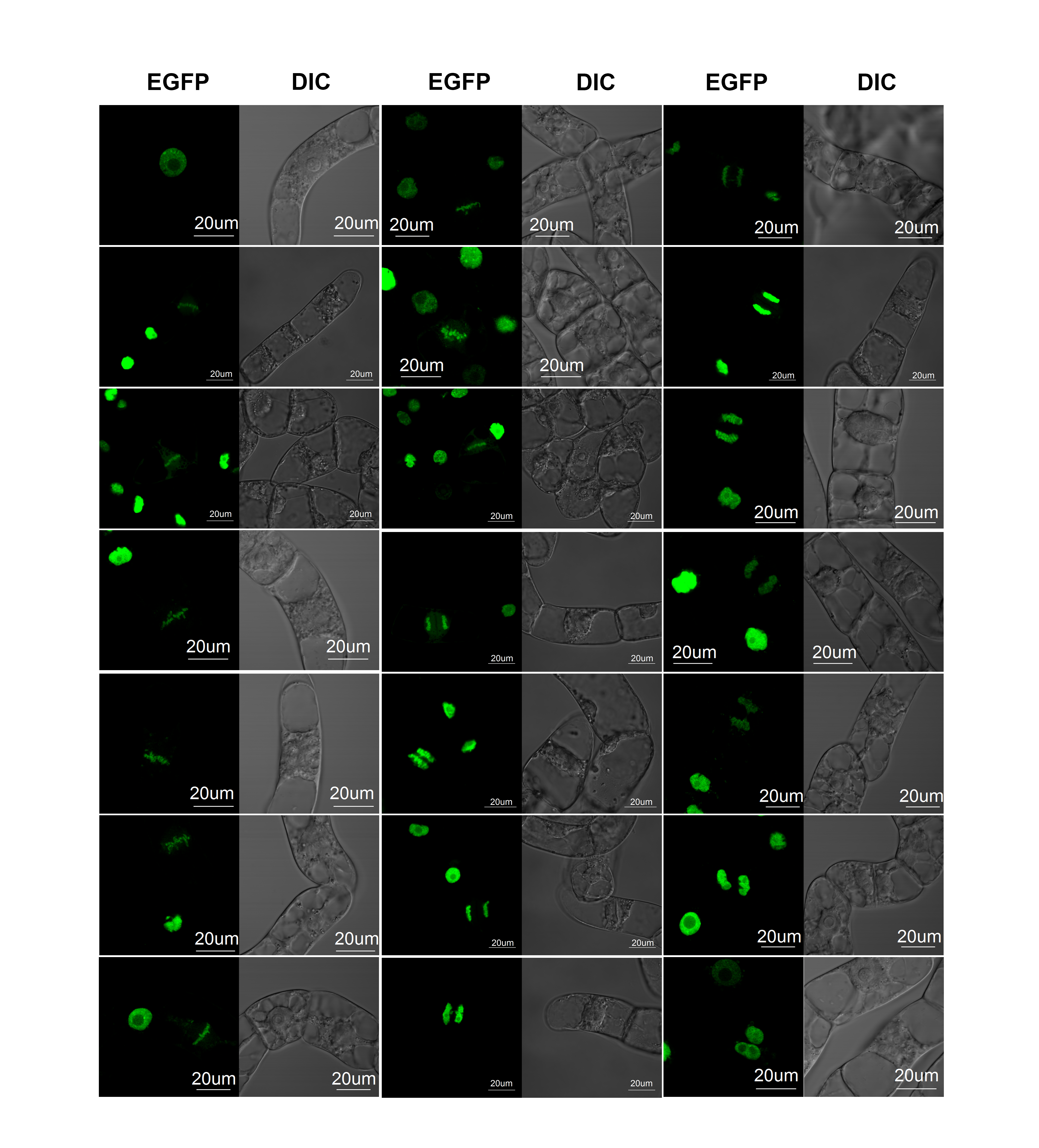

Supplement: S2 Fig — Odd columns showed the EGFP images and the even columns showed the DIC images. (TIF) [file pone.0135033.s002.tif]

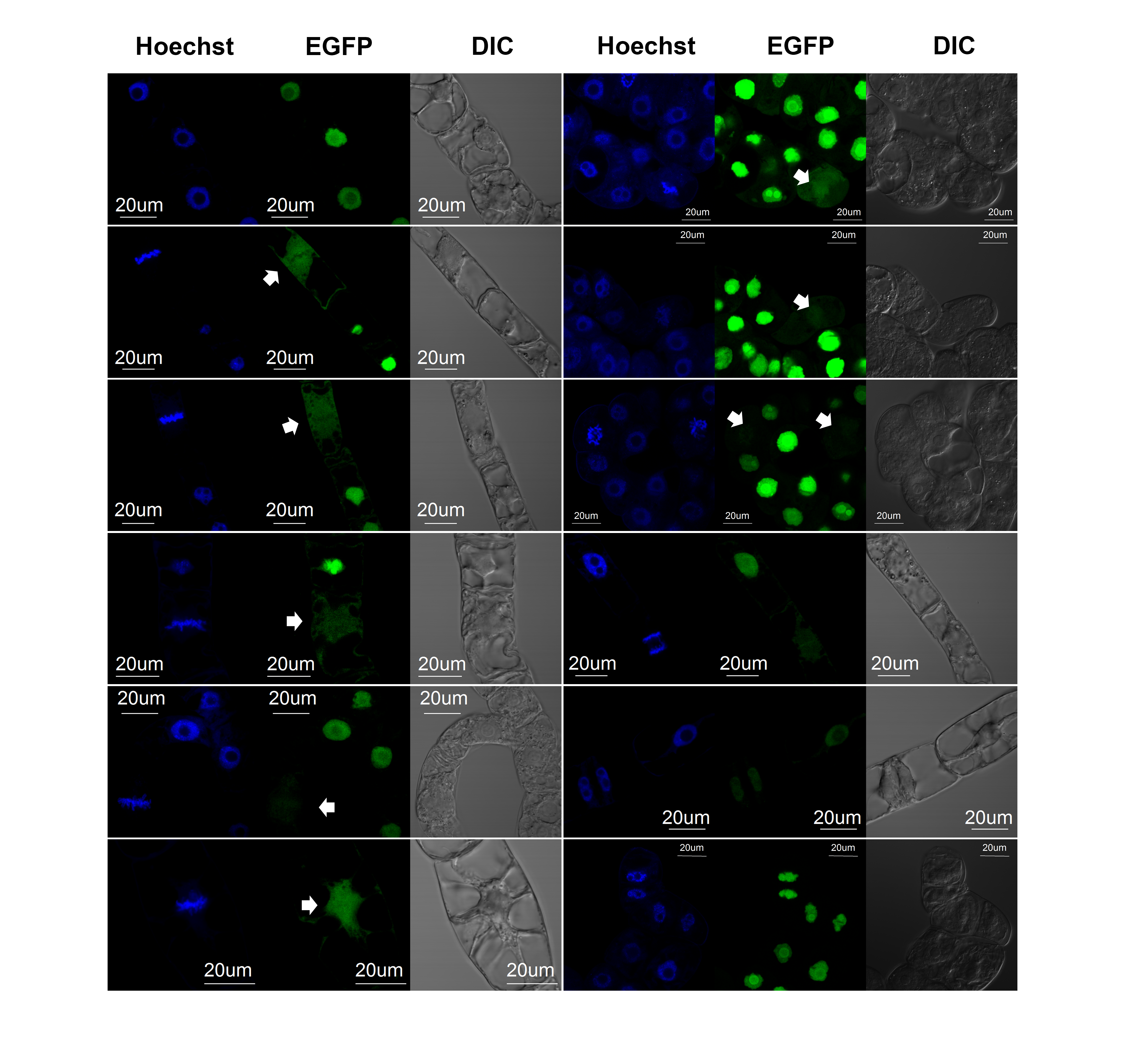

Supplement: S3 Fig — Columns 1 and 4 showed the Hoechst staining images, columns 2 and 5 showed the EGFP images, and columns 3 and 6 showed the DIC images. (TIF) [file pone.0135033.s003.tif]

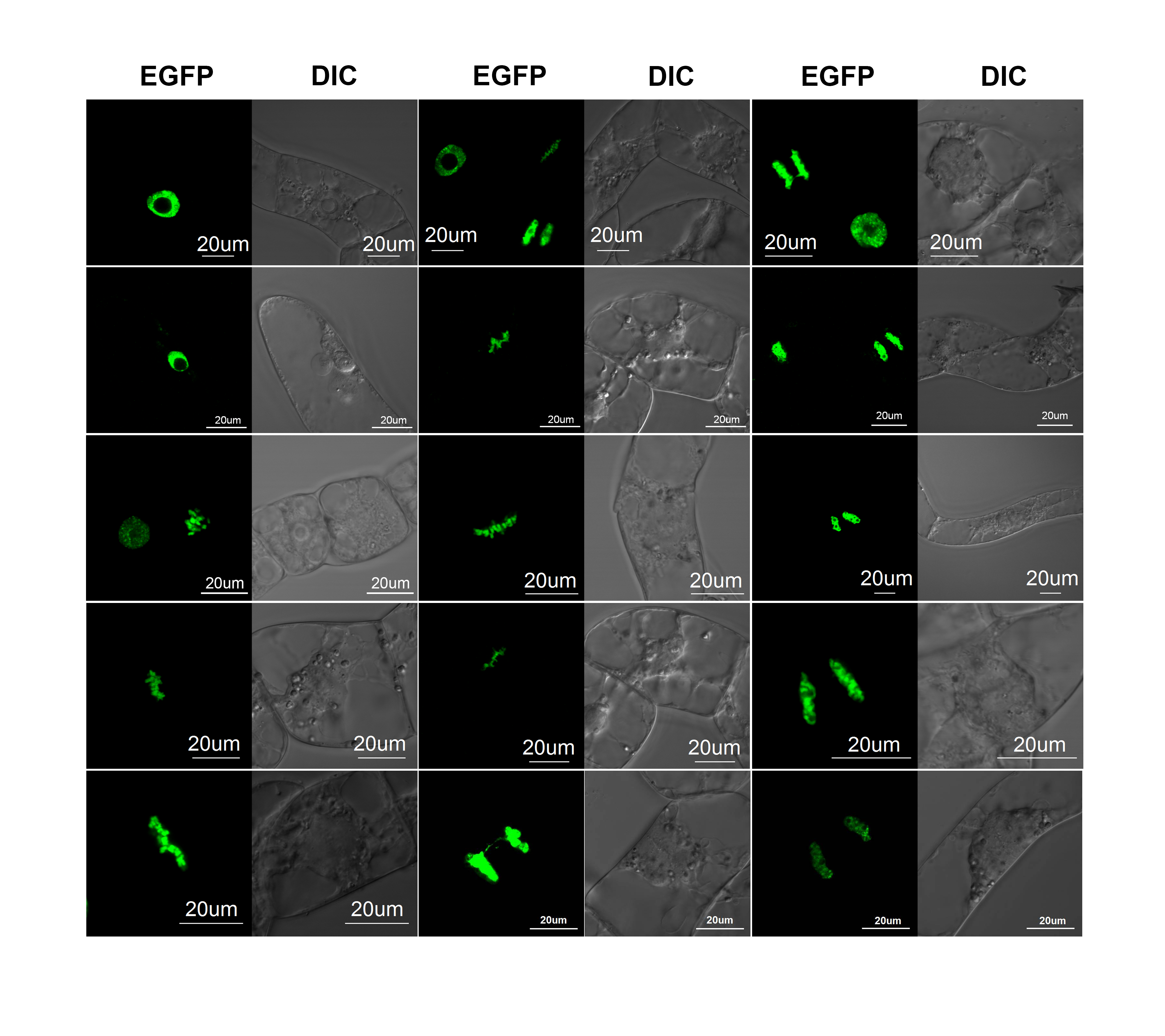

Supplement: S4 Fig — Odd columns showed the EGFP images and the even columns showed the DIC images. (TIF) [file pone.0135033.s004.tif]

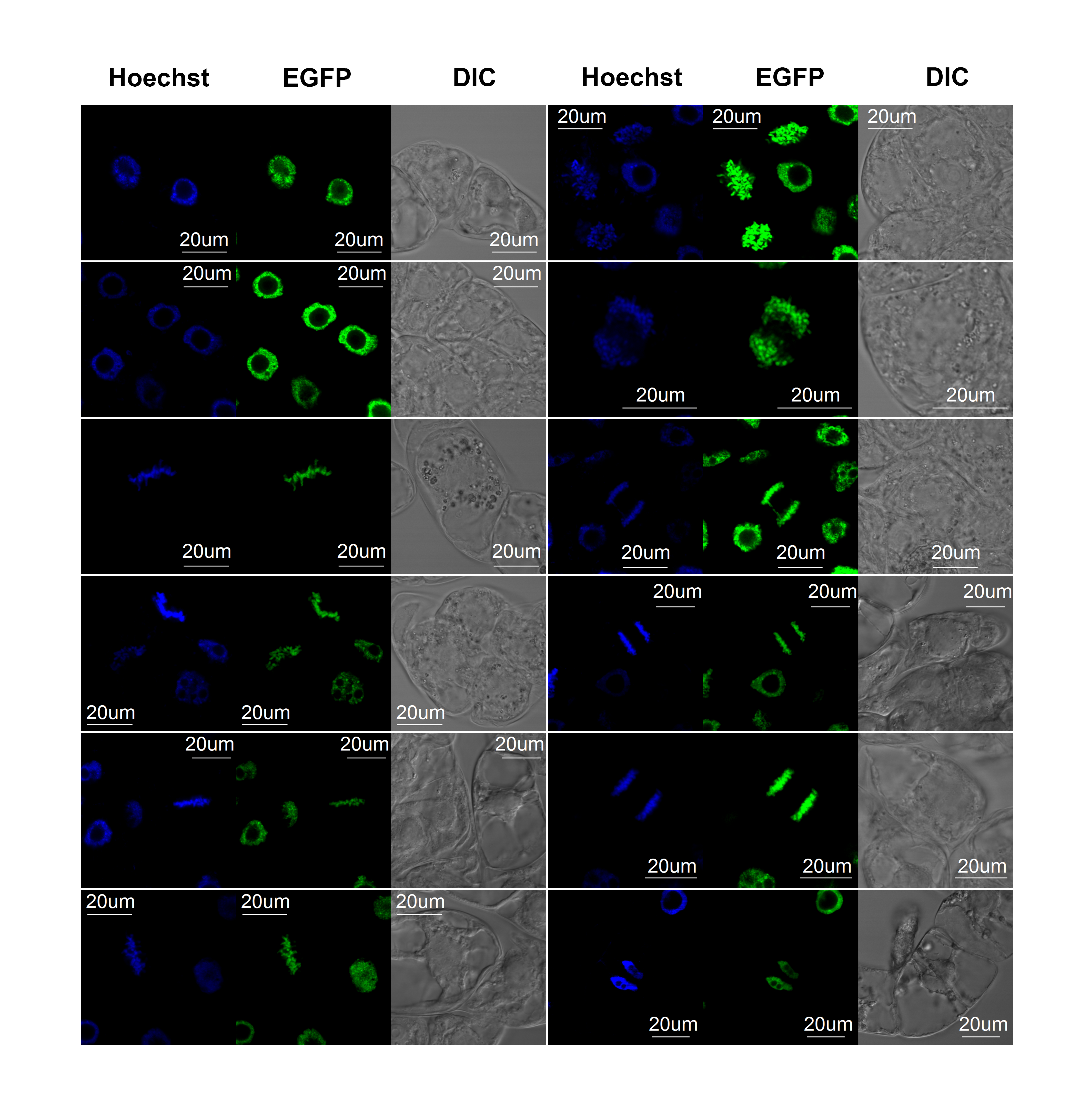

Supplement: S5 Fig — Columns 1 and 4 showed the Hoechst staining images, columns 2 and 5 showed the EGFP images, and columns 3 and 6 showed the DIC images. (TIF) [file pone.0135033.s005.tif]

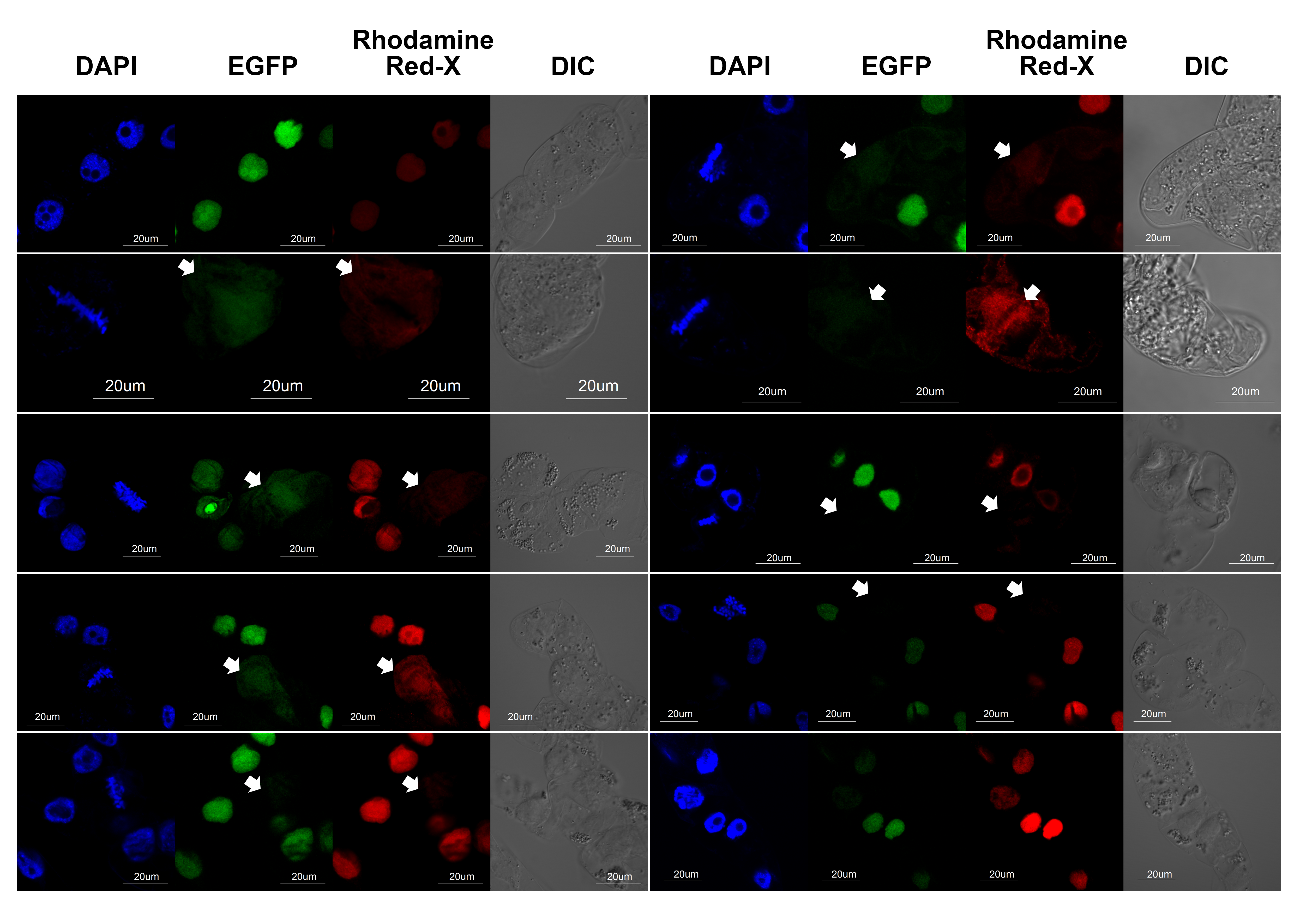

Supplement: S6 Fig — Columns 1 and 5 showed the DAPI staining images, columns 2 and 6 showed the EGFP images, columns 3 and 7 showed the Rhodomine Red-X images, and columns 4 and 8 showed the DIC images. (TIF) [file pone.0135033.s006.tif]

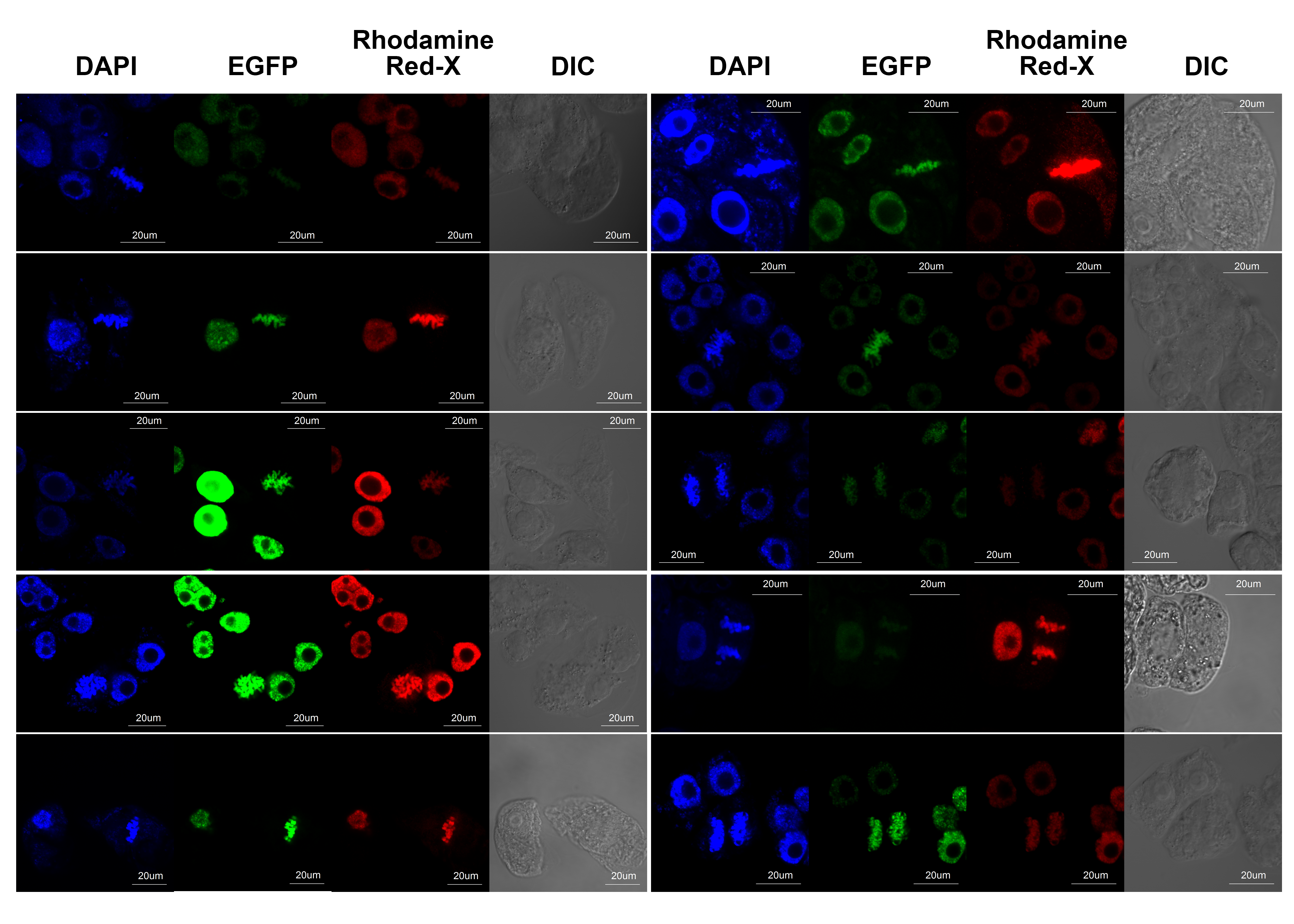

Supplement: S7 Fig — Columns 1 and 5 showed the DAPI staining images, columns 2 and 6 showed the EGFP images, columns 3 and 7 showed the Rhodomine Red-X images, and columns 4 and 8 showed the DIC images. (TIF) [file pone.0135033.s007.tif]
